# Midwives’ experiences of professional learning when practicing collegial midwifery assistance during the active second stage of labour: data from the oneplus trial

**DOI:** 10.1186/s12884-024-06499-8

**Published:** 2024-04-18

**Authors:** Helena Tern, Malin Edqvist, Christine Rubertsson, Maria Ekelin

**Affiliations:** 1 Department of Health Sciences, Faculty of Medicine, Lund University, P.O. Box 117, Lund, SE-221 00 Sweden; 2 Clinical Epidemiology Division, Department of Medicine Solna, Karolinska Institutet, Stockholm, Sweden; 3 Department of Women’s Health, Karolinska University Hospital, Stockholm, Sweden; 4 Department of Obstetrics and Gynecology, Skåne University Hospital, Jan Waldenströms gata 47, Malmö, SE-214 28 Sweden

**Keywords:** Learning, Skill acquisition, Feedback, Midwifery, Labour stage, Second stage, Severe perineal trauma

## Abstract

**Background:**

Learning is a lifelong process and the workplace is an essential arena for professional learning. Workplace learning is particularly relevant for midwives as essential knowledge and skills are gained through clinical work. A clinical practice known as ‘Collegial Midwifery Assistance’ (CMA), which involves two midwives being present during the active second stage of labour, was found to reduce severe perineal trauma by 30% in the *Oneplus* trial. Research regarding learning associated with CMA, however, is lacking. The aim was to investigate learning experiences of primary and second midwives with varying levels of work experience when practicing CMA, and to further explore possible factors that influence their learning.

**Methods:**

The study uses an observational design to analyse data from the *Oneplus* trial. Descriptive statistics and proportions were calculated with 95% confidence intervals. Stratified univariable and multivariable logistic regression analysis were performed.

**Results:**

A total of 1430 births performed with CMA were included in the study. Less experienced primary midwives reported professional learning to a higher degree (< 2 years, 76%) than the more experienced (> 20 years, 22%). A similar but less pronounced pattern was seen for the second midwives. Duration of the intervention ≥ 15 min improved learning across groups, especially for the least experienced primary midwives. The colleague’s level of experience was found to be of importance for primary midwives with less than five years’ work experience, whereas for second midwives it was also important in their mid to late career. Reciprocal feedback had more impact on learning for the primary midwife than the second midwife.

**Conclusions:**

The study provides evidence that CMA has the potential to contribute with professional learning both for primary and second midwives, for all levels of work experience. We found that factors such as the colleague’s work experience, the duration of CMA and reciprocal feedback influenced learning, but the importance of these factors were different for the primary and second midwife and varied depending on the level of work experience. The findings may have implications for future implementation of CMA and can be used to guide the practice.

## Introduction

Learning is a continuous, lifelong process, with the workplace seen as an essential arena for professional development [1]. Workplace learning serves as a pathway to enhance professional skills and acquire knowledge, and formal programs are no longer seen as the only method of learning [2]. This is particularly relevant in professions like midwifery, which demand not only theoretical knowledge but also practical skills that are acquired through clinical practice [3]. Learning in health care settings, such as at obstetric units, is characterised by its complexity and is influenced by numerous factors including organisational, structural, and cultural factors [4, 5], where power hierarchies, norms and values can sometimes act as barriers to effective learning [6, 7]. Furthermore, when learning in clinical settings, the ability to build relationships with colleagues and adapt to sometimes demanding environments is essential [8]. It has been demonstrated that, when combined with factors such as open communication, openness to change, and a strong organisational leadership, collaborative learning with colleagues is an important element in a supportive learning environment [9].

Midwives are required to engage in continuing professional development (CPD) [10] and upgrade their skills in accordance with scientific evidence [3]. In addition, previous research implies that certain midwifery skills require more practice to master than is typically offered within the educational program [11, 12]. The Swedish midwifery program is a post-graduate 18- months university education program at an advanced level. It is offered to registered nurses with a bachelor’s degree, and is divided evenly between theoretical education and clinical placements in various areas and settings [13, 14].

Support for early career midwives is crucial for their professional advancement, particularly during the first years of their career [15, 16]. Many find the transition into midwifery to be an overwhelming period since the nature of the position demands a rapid acquisition of skills to ensure efficient job performance [17]. On-the-job experiential learning, where senior midwives provide feedback and identify knowledge gaps, has been shown to enable early career midwives to develop both safety and confidence in their new positions [10], but this kind of support can be hard to obtain in a busy organisation [18]. In addition, there are concerns regarding the challenges of both achieving and maintaining competence, as many midwives are required to rotate between different wards, which leads to them attending fewer births [11]. It has been suggested that collaborating with a colleague during childbirth can increase professional development and learning, and thus be mutually beneficial for both early-career and senior midwives as their learning needs may differ based on their work experience [11, 19, 20].

Swedish standard care involves one primary midwife responsible for handling a normal birth independently [3] with assistance from a nurse assistant. A second midwife is summoned if complications arise during the birth and an obstetrician is called if additional medical attention is required. Many of the obstetric units in Sweden have a senior midwife in charge who has the overall responsibility of overseeing the organisation and coordinating care, including providing support to colleagues when necessary. Essentially, collegial collaboration and support are typically reserved for critical situations or if required for other reasons.

A clinical practice referred to as ‘Collegial midwifery assistance’ (CMA) has shown a reduction of severe perineal trauma (SPT) by 30% [21]. CMA involves an additional midwife being present during the last phase of the second stage of labour with the specific aim of reducing SPT [21]. Taking part in studies that allow midwives to view childbirth from a new perspective has been found to contribute to professional development [20, 22]. However, to date there have been no quantitative studies conducted investigating the primary and second midwives’ experiences of learning while practicing CMA during the active second stage of labour. Previous research has demonstrated that learning is a social process that occurs through interaction between individuals, with reflection and feedback playing crucial roles [23], particularly when learning specific tasks in clinical practice [24]. In addition, a positive correlation between the duration of time spent and the extent of learning achieved has also been reported [25].

Therefore, the primary aim of this study was to investigate the learning experiences ofprimary and second midwives practicing collegial midwifery assistance, taking into account their varying levels of work experience. We also aimed to further explore potential factors that may be associated with midwives’ learning.

## Methods

### Study design and setting

This study applied an observational design utilising data from the *Oneplus* trial. The primary objective was to evaluate the effectiveness of having an additional midwife present during the active second stage of labour, to prevent SPT [21]. Five Swedish obstetric units with annual births rates ranging from approximately 2,800 to 5,000 were included in the *Oneplus* trial. A detailed description of the data collection procedure of the *Oneplus* trial has been provided elsewhere [21, 26], and is presented below in brief.

### Data collection procedure

Data collection took place between December 10, 2018, and March 21, 2020. The midwives included in this study assisted nulliparous women or those with one previous caesarean section who were planning for their first vaginal birth. These women were randomised to receive either standard care (one midwife) or the intervention (two midwives) when they entered the second stage of labour. The primary midwife decided when to summon the second midwife during the active second stage of labour, who was ready to assist the primary midwife and support the woman when required. No specific guidelines were given for the intervention, other than to follow the unit’s established preventive models for SPT. All midwives had the opportunity to participate as either the primary or second midwife on multiple occasions throughout the study.

**Fig. 1:**
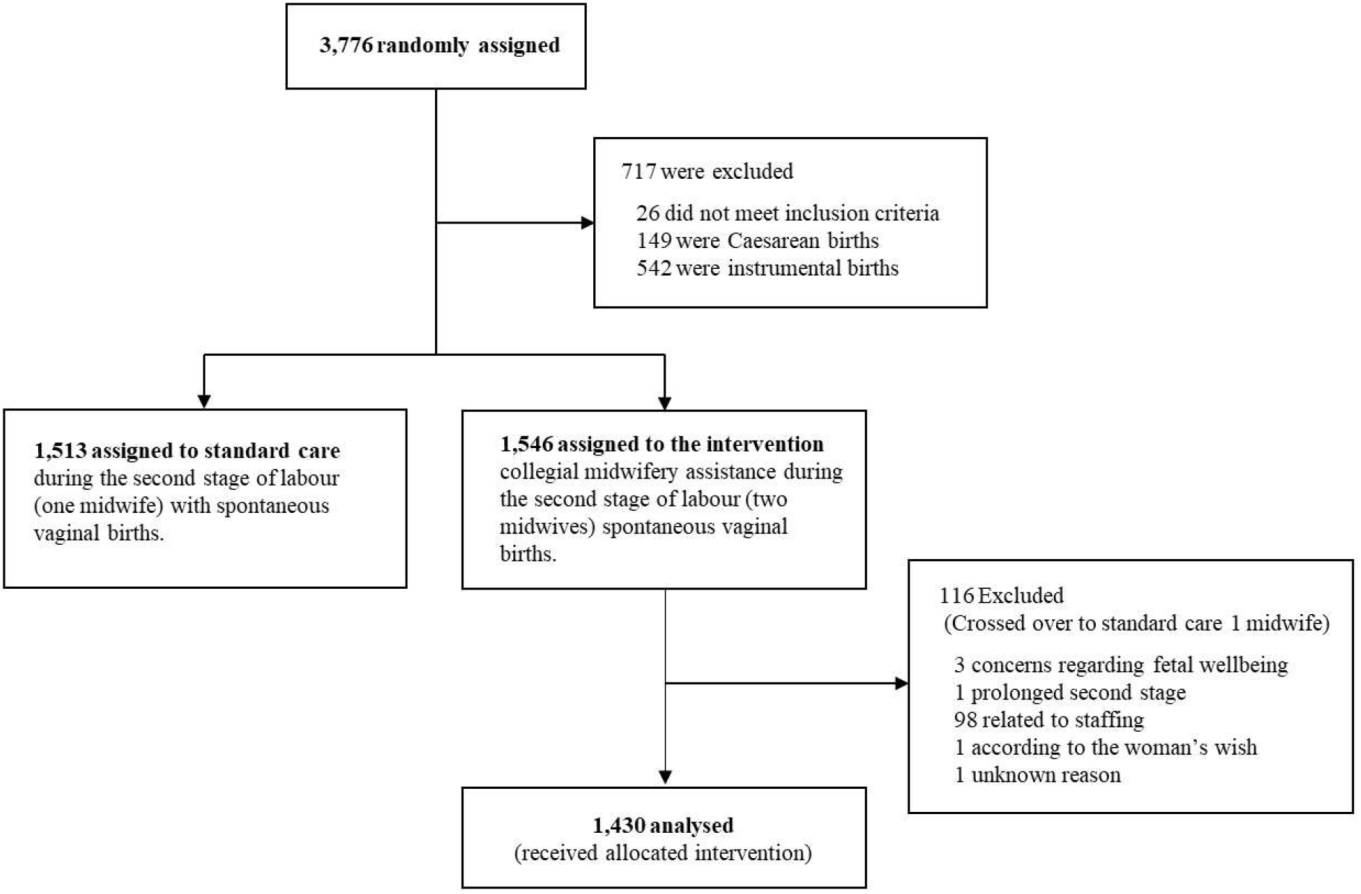
Flowchart of allocation of births to the CMA intervention and exclusions

After each birth involving CMA, the participating midwives completed two clinical report forms (CRFs): one by the primary midwife (CRF no 1), and one by the second midwife (CRF no 2). The CRFs covered multiple aspects relating to the birth, including preventive methods used, the midwives’ experiences of the intervention, the duration of the intervention, and elements pertaining to learning and feedback. The items in the CRFs were study specific and had been developed by the research group in collaboration with clinically active midwives who had experience of CMA. The items underwent a face validation process involving midwives having different levels of work experience to ensure that they would be correctly understood and to enhance their validity. For the purpose of this study, items relating to learning and feedback were primarily utilised. All births that received the allocated intervention (two midwives) and where the woman gave birth spontaneously, were included in the study according to the ‘per protocol’ analysis approach (Fig. 1).

## Measures

### Outcomes

Due to the aim of the study being to investigate the reported learning experiences of both the primary and second midwives when practicing CMA, two outcomes were used. To assess the learning of the primary midwife, the following item from CRF 1 was employed: *‘I learnt something from the second midwife’*. This item was rated on a 4-grade Likert scale and dichotomised to *‘Completely agree’*, ‘*Mostly agree’* and ‘*Partially agree’* as one category and *‘Disagree*’ as another. This dichotomisation was based on the notion that any level of agreement meant that the intervention had contributed to some degree of learning. To assess the learning of the second midwife, the item *‘Have you learnt something from practicing CMA as the second midwife on this particular birth?’* from CRF 2 was used. The response options were yes/no.

### Explanatory variables

Work experience of the primary and the second midwife, reciprocal feedback, and the duration of CMA, were used as explanatory variables in the analyses. These variables were chosen based on the premises that the length of a colleague’s professional work experience influences both the primary and second midwife’s learning [20], that feedback influences learning [27], and that a positive correlation between the duration of the intervention and learning has been observed in a different context [25].

Work experience was categorised from the six original variables [21] into four groups: *<2 years, 2–5 years, 6–20 years, and > 20 years*. The categorisation was considered clinically appropriate while it also allowed for insights into the experiences of the least and most experienced midwives. Further, it was created with consideration of the original data, aiming to mitigate overfitting concerns by ensuring a balanced distribution of observations across the groups. Based on the median time of the CMA interventions and clinical reasoning, the duration of CMA was dichotomised into < 15 min and ≥ 15 min. The variable *‘Reciprocal feedback’* was created by combining two items from CRF 1: *‘I received feedback from the second midwife about my management of the second stage of labour’* and ‘*I gave feedback to the second midwife regarding how I experienced her support/presence/help in the birthing room.* A positive response to both items indicated that there was reciprocal feedback between the midwives. The response options were yes/no.

### Statistical analyses

Descriptive statistics included calculations of frequencies, percentages, medians, and inter quartile ranges (IQR). Proportions were used to analyse learning across groups of work experience for both the primary and second midwife in relation to the explanatory variables. The proportions were derived by dividing the count of reported instances of professional learning during births by the total number of births within each respective group. A 95% confidence interval was established using Jeffrey’s method [28].

In the secondary analysis, separate univariable and multivariable logistic regression models were employed to assess the learning reported by the primary and second midwives. This approach was based on the assumption that learning varies depending on if the midwife acts as the primary or the second midwife. Furthermore, since learning varies depending on work experience, the models were stratified by work experience categorisation as outlined above, and separate logistic regressions were conducted for each of these subgroups to explore interaction effects. Work experience of less than two years was used as the reference group. The crude and adjusted odds ratios were calculated with 95% confidence intervals. All explanatory variables were added simultaneously in the models and were further adjusted for the study site. All statistical analyses were conducted using IBM SPSS Statistics for Windows, Version 28.0 (IBM Corp, Armonk, New York).

## Results

Of the 3,776 women who were randomised in the Oneplus trial, 3,059 gave birth spontaneously [21]. Of those, 1,546 births were randomised to the intervention, and 1,430 received the allocated intervention so were included in the present study (Fig. 1). The median duration of CMA was 15 min (Table 1). In 47% of the births, the primary midwife reported having learned something new to some extent (Table 2). Similarly, the second midwives reported new learning in 38% of the births. In 70% of the births the primary and second midwives engaged in reciprocal feedback.

**Table 1:** Details of the births performed with the CMA intervention

	n (%)
Births with CMA intervention	1,430 (100.0)
Onset of labour	
Spontaneous onset	1,048 (73.3)
Induction of labour	382 (26.7)
Second stage of labour^*^	
Time second stage (minutes), median (IQR)	102 (57–169)
Missing data	2 (0.1)
Active second stage of labour (minutes) median (IQR)	35 (24–52)
Missing data	40 (2.8)
Choice of Second midwife†	
Chosen by the primary midwife	326 (22.8)
First midwife available	481 (33.6)
The second midwife nominated herself	88 (6.2)
Being the midwife in charge (coordinator)	360 (25.2)
Missing data	175 (12.2)
Work experience – primary midwife^§‡^	
< 2 years	485 (33.9)
2–5 years	327 (22.9)
6–20 years	319 (22.3)
> 20 years	293 (20.5)
Missing data	6 (0.4)
Work experience – second midwife^†‡^	
< 2 years	236 (16.5)
2–5 years	262 (18.3)
6–20 years	436 (30.5)
> 20 years	453 (31.7)
Missing data	43 (3.0)
Duration of CMA^†^	
Duration of CMA (minutes), median (IQR)	15 (10–20)
< 15	628 (43.9)
≥ 15	710 (49.6)
Missing data	92 (6.4)

^*^ Begins when the cervix is fully dilated and concludes with the birth of the baby

^†^ Reported by the second midwife

^‡^ All midwives could participate as either the primary or second midwife on multiple occasions

^§^ Reported by the primary midwife

**Table 2:** Details of reported professional learning, feedback, and reflection from the primary and second midwives when practicing CMA in the 1,430 births

	n (%)
The primary midwife reported acquiring new knowledge during the intervention^*^	
Yes^†^	672 (47.0)
No	719 (50.3)
Missing data	39 (2.7)
The second midwife reported acquiring new knowledge during the intervention^‡^	
Yes	546 (38.2)
No	797 (55.7)
Missing data	87 (6.1)
Reciprocal feedback between the primary and second midwife^*^	
Yes	998 (69.8)
No	351 (24.5)
Missing data	81 (5.7)
The second midwife provided feedback when not satisfied with the primary midwife’s technique to prevent SPT^‡^	
Yes	137 (9.6)
No	61 (4.3)
Missing data	80 (5.6)
The second midwife got feedback how the primary midwife experienced her presence^‡^	
Yes	738 (51.6)
No	626 (43.8)
Missing data	66 (4.6)

^*^ Reported by the primary midwife

^†^ Dichotomised from a four-point Likert scale. Yes = ‘Completely agree,’ ‘Mostly agree’ or ‘Partially agree’; No = ‘Disagree’

^‡^ Reported by the second midwife

The length of work experience of the midwives had an impact on reported learning experiences with primary midwives with less work experience reporting new learning more frequently (Table 3), ranging from 76% (< 2 years, 95% CI 0.72–0.80) to 22% (> 20 years, 95% CI 0.17–0.27). A similar pattern was seen for the second midwives where those with the least experience reporting learning in 61% of the births (95% CI 0.55–0.67) and those with the most experience reporting learning in 26% of the births (95% CI 0.22–0.30).

The work experience of the colleague influenced the learning for both the primary and second midwives. The figures revealed that learning was reported in around 35% of cases if the colleague had a work experience of less than two years. The highest rates of learning were seen when the colleague had 6–20 years of work experience, with a slightly higher rate among the primary midwives; 53% (95% CI 0.48–0.58) vs. 45% (95% CI 0.39–0.50) (Table 3). Both primary and second midwives reported increased learning if reciprocal feedback occurred and if the intervention lasted for 15 min or longer.

**Table 3:** Proportions of learning outcomes when practicing CMA based on various explanatory variables in the 1,430 births

	Primary midwife learnt something new during the intervention					Second midwife learnt something new during the intervention				
	*N* = 1,430	n	Proportion	95% CI Lower	95% CI Upper	*N* = 1,430	n	Proportion	95% CI Lower	95% CI Upper
Work experience - primary midwife										
< 2 years	476	362	0.76	0.72	0.80	453	158	0.35	0.30	0.39
2–5 years	322	138	0.43	0.37	0.48	299	128	0.43	0.37	0.48
6–20 years	314	110	0.35	0.30	0.40	306	137	0.45	0.39	0.50
> 20 years	278	61	0.22	0.17	0.27	283	122	0.43	0.37	0.49
Work experience - second midwife										
< 2 years	227	78	0.34	0.28	0.41	230	140	0.61	0.55	0.67
2–5 years	258	117	0.45	0.39	0.51	252	120	0.48	0.41	0.54
6–20 years	423	225	0.53	0.48	0.58	418	168	0.40	0.35	0.45
> 20 years	447	228	0.51	0.46	0.56	435	114	0.26	0.22	0.30
Reciprocal feedback between the primary and second midwife										
Yes	989	542	0.55	0.52	0.58	946	422	0.45	0.41	0.48
No	349	113	0.32	0.27	0.37	330	105	0.32	0.27	0.37
Time CMA ≥ 15 min										
Yes	696	436	0.63	0.59	0.66	679	324	0.48	0.44	0.51
No	620	193	0.31	0.27	0.35	614	202	0.33	0.29	0.37

**Fig. 2:**
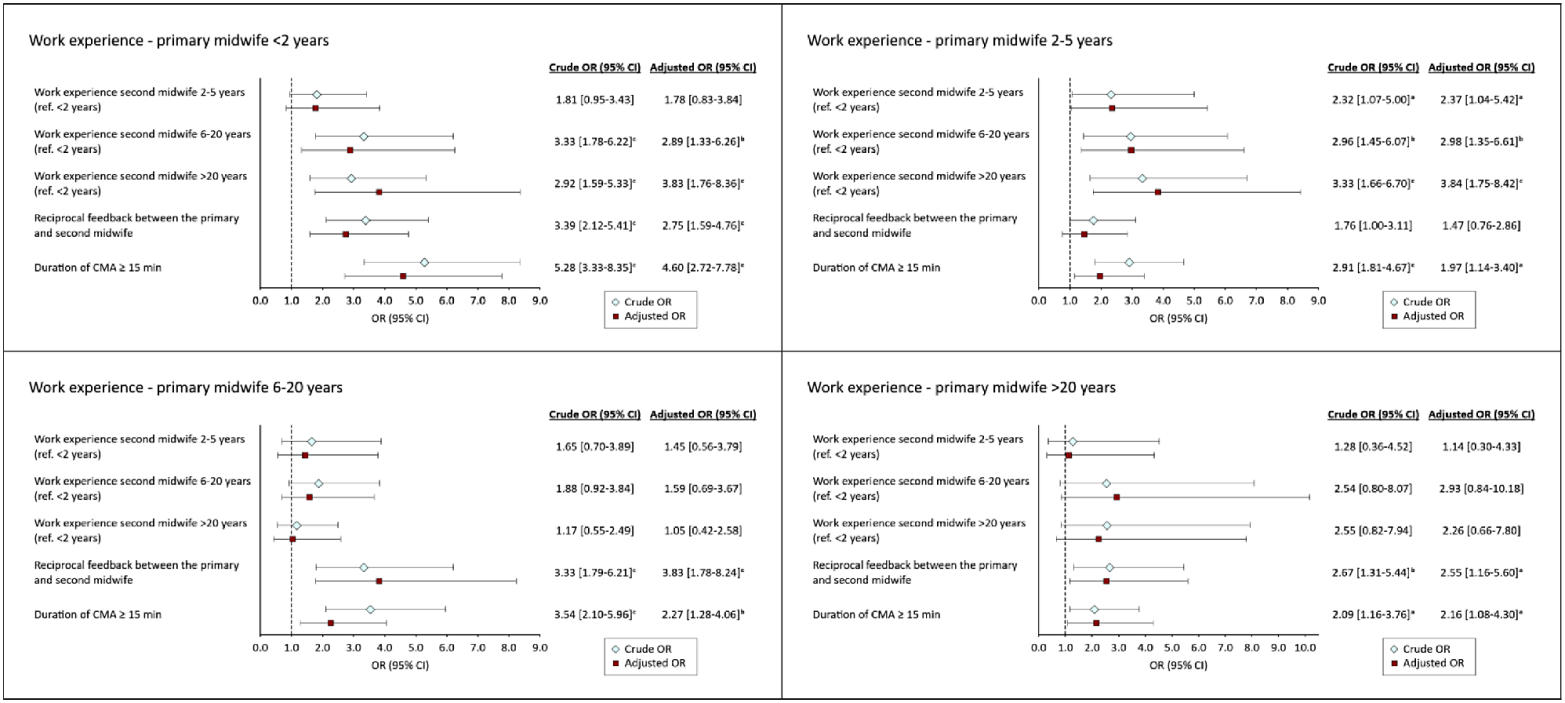
Associations between explanatory variables and the primary midwife’s learning outcomes when practicing CMA, stratified by work experience. Adjusted for study site and explanatory variables.^a^*p* < 0.05. ^b^<0.01. ^c^*p*<0.001

When stratified by the work experience of the primary midwife (Fig. 2), it was observed that reciprocal feedback was beneficial for learning across all groups of primary midwives, with the exception of those with 2–5 years of experience. A similar pattern was seen when the duration of the intervention was 15 min or longer, particularly among the least experienced midwives (aOR 4.60, 95% CI 2.72–7.78). The colleague’s level of experience was also found to be of importance for learning among the less experienced midwives (0–5 years), however, this was not the case among mid- to late-career midwives.

**Fig. 3:**
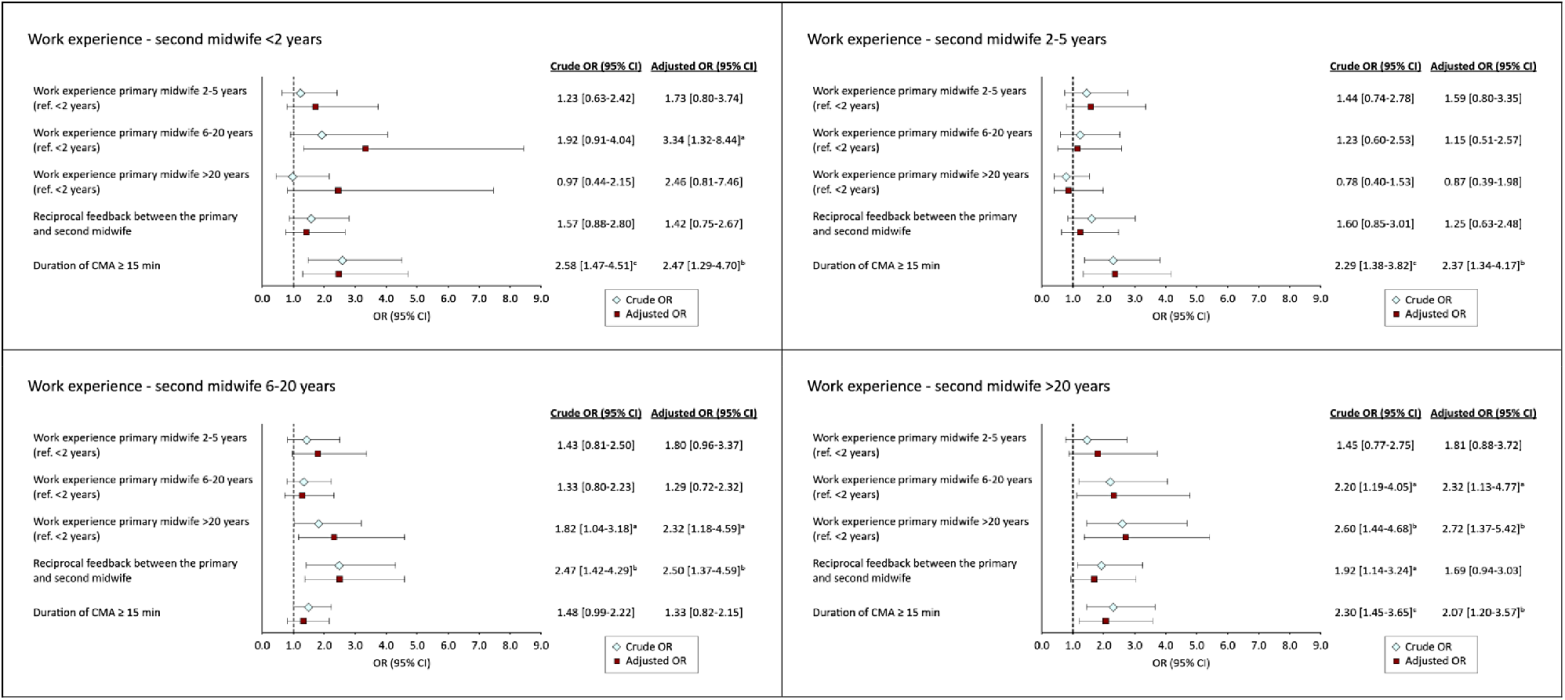
Associations between explanatory variables and the second midwife’s learning outcomes when practicing CMA, stratified by work experience. Adjusted for study site and explanatory variables. ^a^*p* < 0.05. ^b^<0.01. ^c^*p*<0.001

In comparison, among the mid- to late-career second midwives, a colleague with extensive experience was positively associated with learning (Fig. 3). Moreover, the duration of the intervention of 15 min or more was associated with the outcome for the second midwives across all groups, with the exception of those with 6–20 years of work experience. The impact of reciprocal feedback on learning was found to be of less importance when acting as a second midwife, and it was only observed as an influencing factor among those with 6–20 years of work experience (aOR 2.50, 95% CI 1.37–4.59).

## Discussion

The major finding in the current study was that the CMA intervention provided a platform for learning across all groups of work experience, regardless of whether the midwife was in the primary or second midwife role. It was found that if the primary midwife had less than two years of work experience, learning was reported in approximately three out of four births. For the most experienced group (> 20 years of experience), it was reported in just over one in five births. The corresponding results for the most junior second midwives showed that they reported learning in more than three out of five births, whereas the most senior second midwives reported learning in more than one in four births.

Although midwives with less experience reported the highest rates of learning, implying that CMA is particularly beneficial for this group, the study’s findings also confirmed that even the most experienced midwives reported new learning, whether in the role of the primary or second midwife. This is in line with previous qualitative findings, indicating that CMA has the potential to provide lifelong learning opportunities [20]. This is significant because traditionally the focus on professional development has predominantly been on junior midwives, possibly resulting in lack of support for continuing education among late-career midwives [29].

In Sweden, where midwives have traditionally worked independently in the birthing room [30] usually only collaborating with colleagues when the situation demanded it, the introduction of CMA involves a shift in working practices. This change could give senior midwives the opportunity to attend and observe normal uncomplicated births on a daily basis, and not just situations where they are expected to intervene and solve complications.

This study did not investigate *what* the midwives learnt, but midwives have previously reported knowledge transfer occurring in several areas including manual perineal protection, communication skills, and the interpretation of cardiotocography (CTG) results when practising CMA [20]. It has been shown that both teaching and acquiring knowledge during the second stage of labour, while concurrently ensuring the wellbeing of the baby, can be demanding [31]. Therefore, it is interesting that several of the components that make this particular stage complex, such as supporting the birthing woman and protecting the perineum, correspond with what the midwives in the above-mentioned study reported that they learnt when practicing CMA [20]. Although there is a potential risk of being improperly instructed or misguided when learning from a colleague, this may be less likely to occur given the nature of the intervention, where midwives are exposed to births managed by various colleagues and are also given the opportunity to receive and provide feedback. Moreover, the multifaceted nature of the intervention, where midwives are exposed to births managed by various colleagues, aligns with Albert Bandura’s Social Cognitive Theory [32] emphasising the role of diverse observational experiences. Bandura contends that individuals are selective in their observational learning, driven by motivational processes. In this context, the exposure to a range of colleagues provides midwives with an extensive repertoire of practices to observe, allowing them to discern and adopt behaviours that align with their professional motivations.

The study found a positive association between the duration of CMA and reported learning, with CMA lasting 15 min or more being positively associated with reported learning, regardless of the midwife’s level of work experience and both for the primary and second midwife. This is in accordance with the ‘*Time on task hypothesis’*, which posits that learning is a function of time, i.e. that the duration of engagement directly influences the learning outcomes in terms of knowledge acquisition and skill development [33]. While there is evidence to support this, it is inconclusive and available data yield inconsistent findings [25]. Our models revealed that the duration for CMA had the most prominent impact on the most junior midwives. This could be explained in part by the social aspect of learning [24], which involves building new relationships and adapting to new environments in the midwives’ transitions [8]. Further, according to Schön [34] learning involves reflection *in-action*, meaning that learning involves being aware of and adapting during the actual performance. For midwives with less experience, this reflection process may require more time. Additionally, when considering the perspective of second midwives, an extended duration allows for the exploration of a more diverse range of impressions, thereby facilitating comprehension of the situation through acquiring more information about the woman giving birth, the birth itself, and the primary midwives’ line of reasoning.

Feedback is a fundamental component of learning, and there is evidence to support its vital part in the learning process [23, 27]. However, since feedback can be both positive and negative, it can evoke mixed emotions in individuals [35]. Some may perceive it as threatening to receive [36], and it can also be difficult to provide when there is lack of established strategies for delivering feedback [37]. In addition, it has been shown that the manner in which feedback is delivered plays a significant role in how it is perceived [35, 36]. For instance, internal feedback i.e. giving the opportunity for self-assessment prior to receiving external feedback from a colleague can enhance receptiveness [36]. In addition, personal traits such as an individual’s motivation and desire to receive feedback have been shown to be correlated with how the feedback is perceived [35]. Although it was found that reciprocal feedback occurred in 70% of the births included in this study, the results indicate that the importance of feedback varied among the primary and second midwives, and across different levels of work experience. These findings underscore the complexity of learning when practicing CMA, where a range of circumstances can influence learning outcomes.

Estimations indicate that in the future there will be a shortage of midwives in Sweden that is likely to last for an extended period of time [38]. This is primarily due to retirements, which will result in loss of competence [38]. However, a recent report has indicated that there is in fact an adequate number of midwives in Sweden, but retaining them within the field of intrapartum care is the critical challenge [39]. This is particularly relevant to early career midwives due to the current challenging work conditions [18], as beginners are especially vulnerable with their theoretical knowledge but limited clinical experience [17]. An integrated use of CMA could potentially help ease their transition into professional development. CMA may enable them to both be guided through birth scenarios by colleagues and be exposed to a variety of births, which, in turn, can expedite their skill acquisition and experience, as attaining competence in midwifery is a complex and lengthy process [11, 31].

### Strengths and limitations

A major strength in this study is that data were collected prospectively as part of a randomised trial [26] and included data from both the primary and second midwives, enabling a thorough investigation into learning from both perspectives. Furthermore, the stratified analyses conducted in the study rendered a nuanced picture of how midwives experienced learning in each subgroup. However, while informative, the stratified models resulted in smaller subsets of data with less observations in each subgroup, thereby reducing the number of explanatory variables that could be used without risking overfitting the models [40].

Further limitations include the variability in the outcome variables used for the primary and second midwives, which were originally coded slightly differently and thereby lack consistency. The primary midwife’s outcome was based on a Likert scale that was dichotomised during analysis, whereas the second midwife’s outcome was originally recorded as yes/no alternatives. This inconsistency needs to be considered when comparing and interpreting the results. The study also lacks data regarding how many times each of the midwives participated in CMA. However, it is unlikely that the results were affected by bias due to the large number of births and the fact that all midwives working in each unit were allocated women who were included in the study and participated in both the role of primary and second midwife. Despite this, this lack of information should be considered as a limitation.

## Conclusion

The study provides evidence that CMA has the potential to contribute to professional learning both for primary and second midwives, regardless of their levels of work experience. We found that factors such as the colleague’s work experience, the duration of CMA, and the presence of reciprocal feedback influenced learning. However, the importance of these factors varied between primary and second midwives and also depended on their own level of work experience. The findings may have implications for future implementation of CMA and can serve as a guide for the practice.

## Data Availability

As the data used in this study is not covered by the ethical approval for public sharing, it will not be publicly accessible. However, researchers can upon reasonable requests, get access to anonymised data from the corresponding author.

## Abbreviations

CMA: Collegial midwifery assistance
SPT: Severe perineal trauma
CRF: Clinical report form
CTG: Cardiotocography
